# Effect of Moist Cooking Blanching on Colour, Phenolic Metabolites and Glucosinolate Content in Chinese Cabbage (*Brassica rapa* L. subsp. *chinensis*)

**DOI:** 10.3390/foods8090399

**Published:** 2019-09-08

**Authors:** Millicent G. Managa, Fabienne Remize, Cyrielle Garcia, Dharini Sivakumar

**Affiliations:** 1 Phytochemical Food Network Research Group, Department of Crop Sciences, Tshwane University of Technology, Pretoria 0183, South Africa; 2 Université de La Réunion, UMR C-95 QualiSud, 97715 Saint-Denis, Reunion, France

**Keywords:** Brassica vegetables, bioactive compounds, postharvest processing, kaempferol, sinigrin

## Abstract

Non-heading Chinese cabbage (*Brassica rapa* L. subsp. *chinensis*) is a widely consumed leafy vegetable by the rural people in South Africa. Traditional blanching methods (5%, 10% or 20% lemon juice solutions in steam, microwave treatments and hot water bath at 95 °C) on the changes of colour properties, phenolic metabolites, glucosinolates and antioxidant properties were investigated in this study. Blanching at 95 °C in 5% lemon juice solution maintained the chlorophyll content, reduced the difference in colour change ∆E, and increased the total phenolic content and the antioxidant activities (ferric reducing-antioxidant power assay (FRAP) and Trolox equivalent antioxidant capacity (TEAC) assay). The highest concentration of kaempferol-dihexoside, kaempferol-sophoroside, kaempferol hexoside, and ferulic acid was noted in samples blanched in 5% lemon juice, at 95 °C. However, concentrations of kaempferol *O*-sophoroside-*O*-hexoside was highest in raw leaf samples. Supervised Orthogonal Projections to Latent Structures Discriminant Analysis (OPLS-DA) and the UPLC-MS and chemometric approach showed the acid protocatechuoyl hexose unique marker identified responsible for the separation of the blanching treatments (5% lemon juice at 95° C) and raw leaves. However, other unidentified markers are also responsible for the separation of the two groups (the raw leaves and the hot water moist blanched samples) and these need to be identified. Blanching at 95 °C in 10% lemon solution significantly increased the glucosinolate sinigrin content. Overall blanching at 95 °C in 5% lemon juice solution can be recommended to preserve the functional compounds in Nightshade leaves.

## 1. Introduction 

Non-heading Chinese cabbage (*Brassica rapa* L. subsp. *chinensis*), a leafy vegetable, is widely consumed in Venda, Limpopo Province, South Africa [1]. Chinese cabbage is an indigenous African leafy vegetable and it is grown in smallholder cropping systems or in-home gardens. Since the indigenous African leafy vegetable is an inexpensive source of dietary minerals, trace elements and antioxidant phytochemicals, it can be introduced in diet diversification programmes. Chinese cabbage leaves contain Ca (1020 g kg^−1^ FW), Fe (26 g kg^−1^ FW) [2], total glucosinolates (10.926 µmol g^−1^ DW) [3], and phytochemicals such as β-carotene (2305 × 10^−5^ g kg^−1^ FW) and kempferol (0.2002 to 0.25 g kg^−1^) [2]. Glucosinolates are the precursors of the isothiocyanates that are responsible for cancer preventative effects [4]. Thus, it has been proven in in vivo and in vitro studies that Brassicaceae species are capable of the detoxification of carcinogens and the prevention thereof due to their antioxidant activities [4]. The isothiocyanates are responsible for their anticancerogenic [5], anti-inflammatory [6] and antidiabetogenic [7] properties. African vegetables are bitter when eaten in raw form [8]. Therefore, traditionally Chinese cabbage is cooked using various cooking methods such as boiling, steaming or moist cooking (blanching). Traditionally cooked Chinese cabbage is consumed as a side dish with a thick starchy maize meal. However, cooked leaves of Chinese cabbage are currently used in Southern African cuisine as a filling for pastries or burgers. While cooking (thermal processing) reduces the bitterness of the Chinese cabbage [9], extensive cooking procedures can affect the composition of the functional compounds and their bioavailability, and as a result this could affect their biological activity and health benefits, more particularly, causing heat-induced myrosinase inactivation and the reduced production of isothiocyanates [9,10]. However, the extent of the loss of isothiocyanates is dependent on the type of processing method and the duration thereof [11]. 

Furthermore, in East African traditional brassica crops, the predominant flavonoid glycosides are monoacylated kaempferol di-, tri- and tetraglycosides such as kaempferol-3-*O*-sinapoyl-sophoroside-7-*O*-diglucoside and, therefore, benefit human health due to their anticancer and anti-inflammatory activities [11]. Therefore, the objectives of this study were to determine the impact of traditionally used moist cooking on colour, phenolic compounds, glucosinolates and antioxidant activity in Chinese cabbage (*Brassica rapa* L. subsp. *chinensis*).

## 2. Materials and Methods

### 2.1. Plant Material

Chinese cabbage (*Brassica rapa* L. subsp. *chinensis*) leaves were obtained from the Tshiombo irrigation scheme in Venda, Limpopo, South Africa. The leaves were harvested at the 8-leaf stage reached after 60 to 95 days of planting [1]. Leaves free from dirt and damage caused by pests or decay were selected. Thereafter, the leave samples (50 g) were washed with tap water and the leaves were subsequently blanched using a hot water bath, microwave and steam according to the methods described below. 

### 2.2. Moist Cooking Treatment

Chinese cabbage leaves (50 g) were subjected to the following blanching treatments;

(i) blanching at 95 °C in water bath [thermostatically regulated water bath (PolyScience, Niles, IL, USA)] for 5 min in water, or in 5%, 10% or 20% lemon juice solutions;
(ii) a microwave treatment (Defy) (household) working at 2450 MHz–900 W for 5 min in water, or in 5%, 10% or 20% lemon juice solutions;
(iii) steaming in stainless steel steamer pot for 5 min in water or in 5%, 10% or 20% lemon juice solutions at 100 °C.

The pH of the 5%, 10% or 20% solutions were 4.2–4.4, 3.3–3.4 and 2.2–2.5, respectively. 

Thereafter the samples from the selected treatments were subjected to a detailed analysis of the antinutritive compounds, total phenols, and phenolic metabolites and antioxidant properties. The selected samples were snap frozen in liquid nitrogen and stored at −80 °C for all the biochemical analyses. The raw snap frozen samples were included as the control in this study. Each treatment had a set of ten replicates.

### 2.3. Chemicals

Acetone hexane, dimethylsulfoxide (DMSO), methanol, acetonitrile, formic acid, chlorogenic acid (≥95%), catechin (≥95%), luteolin (≥95%), epicatechin (≥95%) and rutinn (≥95%), sodium acetate (≥95%), ferulic acid (≥95%), rutin (≥95%), kaempferol *O*-sophoroside-*O*-hexoside (≥95%), myrectin-*O*-arabinoside (≥95%), 2,4,6-tris(2-pyridyl)-1,3,5-triazine, hydrochloric acid (HCl), ferric chloride (FeCl_3_), Trolox, 2,2′-azobis(2-amidinopropane) hydrochloride (ABAP), 2,2′-azinobis(3-ethylbenzothiazoline-6-sulfonate), phosphate, sodium chloride (NaCl), ammonium hydroxide (NH_4_OH), Folin–Ciocalteu reagent, sodium carbonate (Na_2_CO_3_), gallic acid, 1 methoxyglucobrassicin, 4-methoxyglucobrassicin and sinigrin were purchased from Sigma Aldrich, Johannesburg, South Africa. 

### 2.4. Colour Measurement

The colour of the Chinese cabbage leaf was measured using a Minolta CR-400 chromameter (Minolta, Osaka, Japan). In the International Commission on Illumination (CIE) CIE colour system, colour coordinate a* can be related to the red and green colours when it has a positive or negative value. Similarly, colour coordinate b* can be described as a yellow colour when it is positive. The colour changes (∆*E*) were calculated using the following formula [12].

$$E_{\mathrm{ab}}^{*}=\sqrt{{\left(L_{1}^{*}-L_{2}^{*}\right)}^{2}+{\left(a_{1}^{*}-a_{2}^{*}\right)}^{2}+{\left(b_{1}^{*}-b_{2}^{*}\right)}^{2}}$$

where *L*_1_*, *a*_1_*, *b*_1_* are the values for raw sample values. *L*_2_*, *a*_3_*, and *b*_3_* are the values of the sample subjected to different blanching treatments. Measurements were taken at three points on the per replicate and ten replicate samples per treatment were used for the determination of colour changes. 

### 2.5. Chlorophyll

The chlorophyll a (*Chl a*) b (*Chl b*), and total chlorophyll were determined without modifications using leaf samples (0.2 g) ground with 2 mL of acetone and hexane 4:6 (*v*/*v*) and extracted for 2 h. Afterwards, the sample mixture was centrifuged for 10 min at 4 °C (9558× *g*). Thereafter, the resulting supernatant was decanted, and a portion of the solution was measured at 470, 646 and 662 nm (Biochrom Anthos Zenyth 200 Microplate Reader; SMM Instruments, Biochrom Ltd., Johannesburg, South Africa). The *Chl a* and *Chl b* contents were determined according to equations: *Chl a* = 15.65*A*662 − 7.340*A*646 and *Chl b* = 27.05*A*646 − 11.21*A*662. The content of *Chl a* + *Chl b* gives the total chlorophyll content and it was expressed in mg per 100 g on a fresh weight basis [2].

### 2.6. Total Phenol and Predominant Metabolic Profile

#### 2.6.1. Total Phenol Content

Snap frozen Chinese cabbage (0.2 g) was homogenized in 2 mL of 80% methanol (*v*/*v*), and then centrifuged at 10,000× *g* for 10 min at 4 °C using Hermle Labortechnik, Germany. Total phenolic content was determined using the modified method of Singleton, Orthofer and Lamuella-Raventós (1999). An aliquot of 9 μL of supernatant extract was mixed with 109 μL of Folin–Ciocalteu reagent then followed by 180 μL of 7.5% Na_2_CO_3_. The total of the phenolic compounds was calculated using gallic acid and the results were expressed as mg 100 g^−1^ gallic acid equivalents (GAEs) on a fresh weight basis. 

#### 2.6.2. Predominant Metabolic Profile

The detection and quantification of predominant metabolites were carried out using the Quadrupole time-of-flight (QTOF) mass spectrometer (MS) UPLC–Q-TOF/MS (Waters, Milford, MA, USA). The conditions for separation of the phenolic compounds are similar to Ndou et al. [13]. 

Due to the unavailability of the calibration standards for all the compounds, the identification was carried out by means of quantification against the calibration curves set up using chlorogenic acid, catechin, luteolin, epicatechin and rutin as described by Stander et al. [14]. Four different cocktails were made at each level to facilitate the identification of the isomers and compounds with similar elemental formulas as described by Stander et al. [14]. Cocktails were prepared in methanol (50%) in H_2_O containing formic acid (1%) solution. The main peaks in each chromatogram were quantified by setting up the TargetLynx processing method (part of MassLynx). Extracted mass chromatograms were defined for each compound, based on the retention time and accurate mass obtained from the high-resolution mass spectrometer [13]. Due to the unavailability of the calibration standards for all the compounds identified, these were semi-quantitatively measured against calibration curves set up using chlorogenic acid, catechin, luteolin, epicatechin and rutin [13]. Extracted mass-retention time pairs for each compound were defined in the TargetLynx method and the closest eluting calibration compound (chlorogenic acid, catechin, epicatechin, or rutin) was set as the calibration reference compound. A range of calibration standards containing from 1 to 200 mg/L chlorogenic acid, catechin, epicatechin and rutin were injected using the same method of Stander et al. [14] for the samples. The data was then reprocessed using the established TargetLynx method to produce integrated peak areas for each compound, which were then interpolated off the calibration curves for the reference compounds. Based on the masses of the plant material extracted, the volumes of extraction solvent used, and the dilutions employed, the concentrations of the compounds in the plant material was calculated by the TargetLynx software as previously shown by Stander et al. [14].

Chlorogenic acid, catechin, epicatechin ferulic acid, rutin, ranging from 1 to 200 mg/L were injected as calibration standards using the same method of Stander et al. [14]. The data was then reprocessed using the established TargetLynx method to produce integrated peak areas for each compound, which were then interpolated off the calibration curves for the reference compounds. The concentrations of the compounds in the plant material was calculated by the TargetLynx software based on the masses of the extracted plant material, the volumes of extraction solvent used, and the dilutions employed as described previously in our research [13]. 

### 2.7. Total Antioxidant Capacities Were Determined Using the Following Assays

The ferric reducing-antioxidant power assay was executed following the method described by Mpai et al. [15]. Nightshade leaf samples (0.2 g) were homogenized in 2 mL of sodium acetate buffer at a pH of 3.6. The ferric-reducing ability was estimated by mixing a 15 μL aliquot of leaf extract, with 220 μL of FRAP reagent solution (10 mmol L^−1^ 2,4,6-tris(2-pyridyl)-1,3,5-triazine (TPTZ)) acidified with concentrated HCl, and 20 mmol L^−1^ FeCl_3_]. The absorbance was read at 593 nm and the reducing antioxidant power content was calculated using a standard curve of Trolox and expressed μmol Trolox equivalent antioxidant capacity (TEAC) g^−1^ FW. 

For the determination of the ABTS assay, the 2.5 mM 2,2′-azobis (2-amidinopropane) hydrochloride (ABAP) and 20 mM 2,2′-azinobis(3-ethylbenzothiazoline-6-sulfonate) ABTS 2 stock solution in 100 mL of phosphate buffer (100 mM phosphate and 150 mM NaCl, pH 7.4) were mixed and incubated at 60 °C for 6 min without any modifications as described by Egea, Sánchez-Bel, Romojaro, and Pretel [16].

To produce the ABTS radical anion, the mixture was held in darkness for 16 h at 25 °C and afterwards diluted with 0.1 mM phosphate buffer (pH 7.0) to obtain an absorbance at 734 nm (1.1 ± 0.002 units). Thereafter, the radical solution (285 μL) was added to the sample extract (15 μL) and the decrease in absorbance observed at 734 nm for 6 min was used to calculate the Trolox equivalent antioxidant capacity (TEAC). Calibration curves were constructed for each assay using different concentrations (0–20 mg) of Trolox. The antioxidant activity (ABTS assay) was expressed as µmg of TEAC g FW^−1^.

### 2.8. Glucosinolate

Samples were prepared by extracting 5 g of sample with 15 mL of extraction solvent (50% MeOH in 0.1% formic acid). After sonication in an ultrasonic bath for 1 h, the samples were centrifuged at 14,000× *g* for 5 min. A clear sample was transferred to 2 mL glass vials for analysis. A Waters Synapt G2 Quadrupole time-of-flight (QTOF) mass spectrometer (MS) connected to a Waters Acquity ultra-performance liquid chromatograph (UPLC) (Waters, Milford, MA, USA) was used for high-resolution UPLC-MS analysis. Electrospray ionization was used in the negative mode with a cone voltage of 15 V, desolvation temperature of 275 °C, desolvation gas at 650 L h^−1^, and the rest of the MS settings optimized for best resolution and sensitivity. Data were acquired by scanning from *m*/*z* 100 to 1200 *m*/*z* in the resolution mode as well as in the mass spectrometry (MS) E represents collision energy MSE mode. In the MSE mode, two channels of MS data were acquired—one at a low collision energy (4 V) and the second using a collision energy ramp (40–100 V)—to obtain fragmentation data as well. Leucine enkaphalin was used as the lock mass (reference mass) for accurate mass determination and the instrument was calibrated with sodium formate. Separation was achieved on a Waters Acquity BEH (Ethylene-bridged hybrid) C18, 2.1 × 100 mm, 1.7 μm column. An injection volume of 2 μL was used and the mobile phase consisted of 0.1% NH_4_OH in water (solvent A) and acetonitrile containing 0.1% NH_4_OH acid as solvent B. The gradient started at 100% solvent A for 0.3 min and changed to 3% B over 3 min in a linear way. It then went to 28% B at 9 min, followed by 100% B after 9.1 min, with a wash step of 0.9 min at 100% B, followed by re-equilibration to initial conditions for 3 min. The flow rate was 0.3 mL min^−1^ and the column temperature was maintained at 55 °C. Glucosinolates were quantified in a relative fashion against sinigrin, with calibration standards ranging from 10 to 100 mg L^−1^. Other glucosinolates, including 1 and 4-methoxyglucobrassicin, were identified on the basis of accurate mass elemental composition and fragmentation patterns.

### 2.9. Statistical Analysis 

A completely randomized design was adopted with ten replicates per treatment and the experiments were repeated twice. A factorial type (4 × 4 or 4^2^) experiment was conducted, which includes the different types of moist cooking and the control (raw, steam, microwave and hot water bath) and the type of blanching media (water, 5% lemon juice, 10% lemon juice or 20% lemon juice) on the change of colour difference, chlorophyll content. Two-way analysis of variance (ANOVA) was used to analyse the mean differences between different blanching treatments at a significance level of *p* < 0.05. Interaction between “the moist cooking methods” and the “type of blanching media” was investigated in this study for parameters such as colour difference and chlorophyll content. After selecting the best method of moist cooking (hot water bath) the different types of blanching media (water, 5% lemon juice, 10% lemon juice), one-way ANOVA was performed on total phenolic compounds, predominant phenolic compounds, antioxidant activities (FRAP and TEAC assay) and sinigrin content. Means were compared among treatments by the least significant difference (LSD) test with *p <* 0.05 considered to indicate statistical significance. The data were analysed using the Genstat for Windows 13th Edition (2010) (VSN International, Hempstead, UK).

## 3. Results 

### 3.1. Colour Difference, Chlorophyll Content

**Figure 1:**
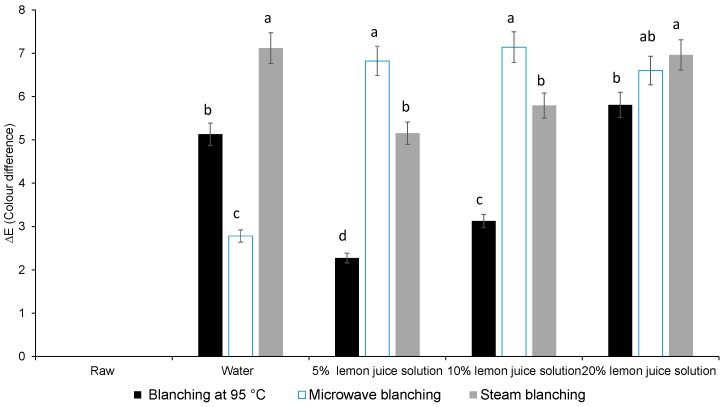
Effect of different types of moist cooking blanching treatments on colour difference (∆*E*) in Chinese cabbage leaves. Bars with the same alphabetic letter per moist cooking treatment are not significantly different at *p* < 0.05).

Moist cooking blanching (dipping) in 5% or 10% lemon juice solution, at 95 °C in a water bath, significantly minimised the difference in colour change (∆*E*) (Figure 1). All the other moist cooking blanching treatments adopted in this study revealed a significantly high difference in colour change (∆*E*) due to the olive brown colour of the leaves (Figure 1). The total chlorophyll content was significantly reduced during steam, hot water bath and microwave blanching when 20% lemon juice solution was used as the blanching medium. 

**Figure 2:**
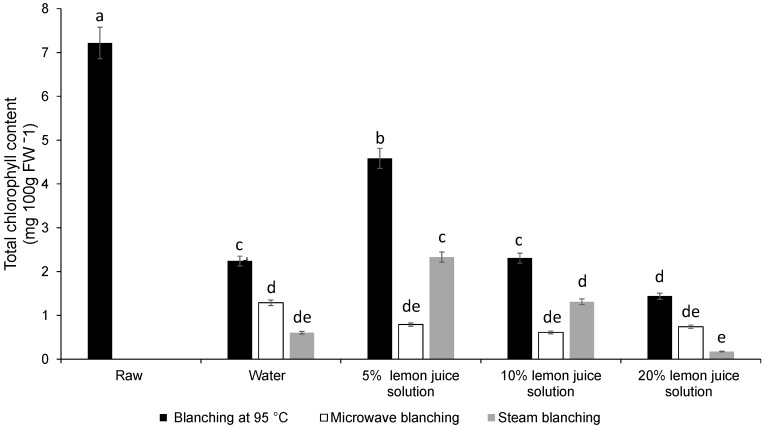
Effect of different types of moist cooking blanching treatments on total chlorophyll content in Chinese cabbage leaves. Bars with the same alphabetic letter are not significantly different at *p* < 0.05).

Similarly, blanching in 5% lemon juice solution, at 95 °C in a water bath, significantly retained the total chlorophyll content followed by the 5% lemon juice solution (water bath) at 95 °C (Figure 2). Overall, microwave and steam blanching in both 10 and 20% lemon solution significantly reduced the total chlorophyll content (Figure 2). 

Therefore, Chinese cabbage leaves blanched in 5% or 10% lemon juice solution and water, as a blanching medium at 95 °C in a hot water bath, were selected for further analysis of phenolic compounds and antioxidant activity.

### 3.2. Total Phenolic Compounds and Phenolic Components

**Figure 3:**
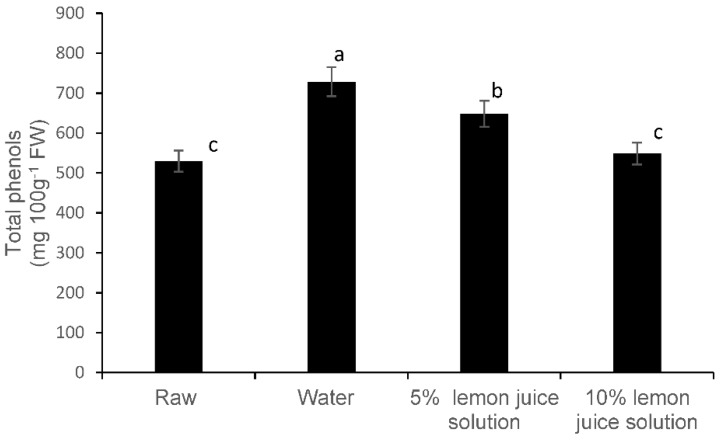
Effect of different types of moist cooking blanching treatments on total phenol content in Chinese cabbage leaves. Bars with the same alphabetic letter are not significantly different at *p* < 0.05.

Blanching in a water bath at 95 °C using water as a blanching medium significantly increased the total phenolic content in Chinese cabbage compared to the raw leaves (Figure 3). However, with an increasing concentration of lemon juice, a declining trend in total phenolic content was noted (Figure 3). When 10% lemon juice was used as blanching medium, the total phenolic content was maintained at similar levels as raw leaves (Figure 3). 

Total ion chromatograms of the Chinese cabbage samples subjected to different blanching treatments and blanching media in the Electrospray ionization (ESI) mode by UPLC–Q-TOF/MS were illustrated in Table 1 and Figure S1. 

**Figure 4:**
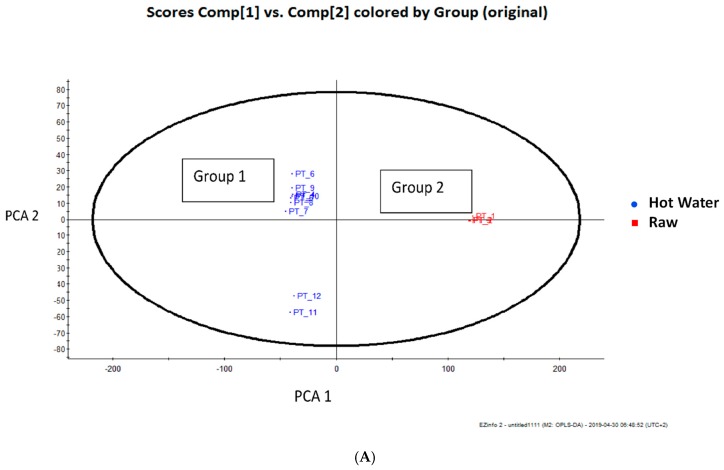

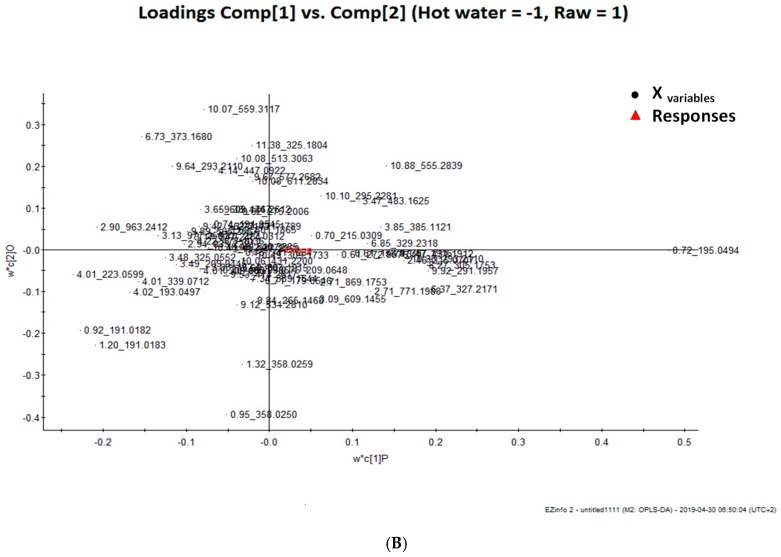
(**A**) Score plot of principal component analysis (unsupervised) based on UPLC–Q-TOF/MS spectra of different moist cooking blanching treatments. Group 1 included the hot water bath blanching at 95 °C using water or 5% or 10% lemon juice as blanching medium for 5 min. Group 2 included the raw leaves. (**B**) Loading of Principal component analysis based on UPLC–Q-TOF/MS spectra of different moist cooking blanching treatments.

**Figure 5:**
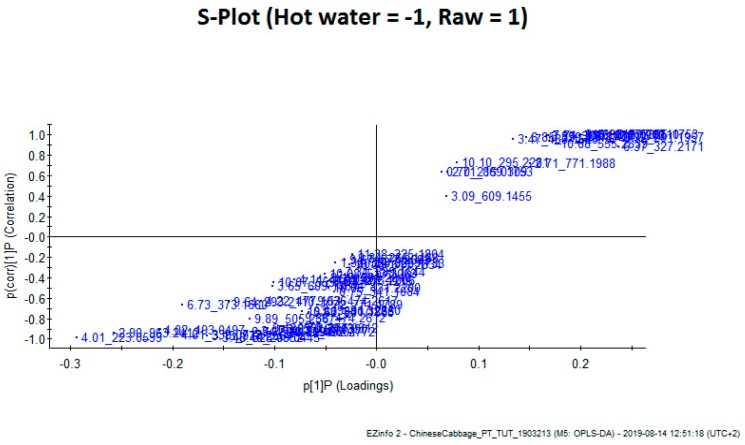
Score plot of orthogonal partial least squares discriminant analysis of ultra-performance liquid–quadrupole time-of-flight (QTOF) mass spectrometer (MS) (UPLC–Q-TOF/MS) spectra of hot water bath blanching treatments and raw samples. Each sample set includes three replicates.

The differences between the phenolic metabolic profiles of the different hot water bath blanching treatments and blanching media compared with that of the raw leaves were evident when using an unsupervised Principal Component Analysis (PCA) approach using the data generated by the UPLC–Q-TOF/MS analysis. Figure 4A showed the PC 1 and PC 2 explaining 41% and 17% of the variance and illustrating good statistical separation among the various adopted moist cooking blanching treatments. The PCA plot, which has three groups based on the metabolites, demonstrated that the blanching treatments influenced the metabolites in Chinese cabbage leaves. Group 1 included the hot water bath blanching at 95 °C using water or 5% lemon juice solution as the blanching medium for 5 min, and Group 2 included blanching using a hot water bath with 10% lemon juice solution as the blanching medium for 5 min (Figure 4A). However, to explain the two groups blanching in a hot water bath and steaming in water or 5% solution of lemon juice and to identify the potential characteristic markers (metabolites) responsible for discrimination between the treatments, supervised Orthogonal Projections to Latent Structures Discriminant Analysis (OPLS-DA) was performed. The potential markers were chosen based on the weightage of their contribution towards the variation and correlation within the data set. This model showed greater reliability and validity (variance recorded at 8.98%) (Figure 4A). In the S-plot, the points are Exact Mass/Retention Time pairs (EMRTs) plotted by covariance (x-axis) and correlation (y-axis) values (Figure 4B). The S-plot helped to identify the EMRT pairs that contributed towards the most significant difference between the raw Chinese cabbage leaves and those subjected to hot water blanching treatment (Figure 4). The loadings from a two-class OPLS-DA model (Hot water vs. Raw) are shown here in an S-Plot format for Raw (Figure 5). The points are Exact Mass/Retention Time pairs (EMRTs) plotted by covariance (x-axis) and correlation (y-axis) values. The upper right quadrant of the S-plot shows those components which are elevated in the control group, while the lower left quadrant shows components elevated in the treated group. The farther along the x-axis, the greater the contribution to the variance between the groups, while, the farther the Y axis, the higher the reliability of the analytical result. Some of the most important EMRTs are tabulated and plotted below. The candidate markers responsible for the observed trend in the S-plot are shown in Table 2 and Table 3. Based on Table 2 and Table 3, the protocatechuoyl—hexose is the only phenolic compound successfully identified as a marker of the difference in the phenolic profiles of raw Chinese cabbage. This compound was not found in the blanched leaves. This was confirmed by the quantitative analysis that showed a disappearance of this compound in hot water blanching. Other unidentified compounds (markers) that were responsible for the observed separation will be identified as part of our future work.

The UPLC–Q-TOF/M analysis helped to identify 10 compounds: gluconic acid (*m*/*z* 195.0493, λ 227), malic acid (*m*/*z* 133.0127), quinic acid (*m*/*z* 191.0181, λ 280), protocatechuoyl—hexose (*m*/*z* 315.0707, λ 306), kaempferol 3-*O*-sophoroside 7-*O*-hexoside (*m*/*z*, λ 265.347), kaempferol-dihexoside (*m*/*z* 609.1463, λ 265.341), kaempferol 3-*O*-sophoroside (*m*/*z* 609.1488 λ 264.340), kaempferol hexoside (*m*/*z* 447.0947, λ 264,350), and myricetin 3-*O*-arabinoside (*m*/*z* 449.0743, λ 364,350), as shown in Table 1 and Figure S1.

The UPLC–Q-TOF/MS quantified phenolic profile obtained for raw Chinese cabbage showed the highest content of quininic acid (209 mg kg^−1^), kaempferol *O*-sophoroside-*O*-hexoside (50.4 mg kg^−1^), ferulic acid (50.3 mg kg^−1^) and protocatechuoyl—hexose (46 mg kg^−1^), followed by kaempferol-dihexoside (8.0 mg kg^−1^), kaempferol hexoside (20.8 mg kg^−1^) and myrectin-*O*-arabinoside (20.3 mg kg^−1^) (Table 4). Different concentrations of lemon juice blanching media affected the concentrations of the phenolic compounds (Table 4). Hot water bath blanching using 10% lemon juice as the blanching medium significantly reduced the concentration of kaempferol *O*-sophoroside-*O*-hexoside, kaempferol-dihexoside, kaempferol-sophoroside, kaempferol hexoside and myrectin-*O*-arabinoside compared to the raw Chinese cabbage samples (Table 4). Therefore, using 10% lemon juice blanching medium at 95 °C must be avoided. However, a 5-fold increase in the quinic acid concentration was noted in samples blanched in water or 5% lemon juice at 95 °C compared with the raw samples (Table 4), whereas, the 10% lemon juice blanching medium at 95 °C showed an 8-fold increase in quinic acid compared to the raw samples (Table 4). The ferulic acid concentration was significantly the highest during blanching in water or 5% lemon juice at 95 °C. Protocatechuoyl hexose was detected in only the raw samples (Table 4). The highest concentrations of kaempferol-dihexoside, kaempferol hexoside were obtained during blanching in 5% lemon juice at 95 °C (Table 4), whereas the highest concentration of kaempferol-dihexoside, kaempferol-sophoroside and kaempferol hexoside was noted in samples blanched in 5% lemon juice at 95 °C (Table 4). The highest concentrations of kaempferol *O*-sophoroside-*O*-hexoside and myrectin-*O*-arabinoside were detected in the raw Chinese cabbage samples (Table 4). The ferulic acid content did not change significantly when water or 5% lemon water was used as the blanching medium (Table 4).

### 3.3. Antioxidant Activity 

**Figure 6:**
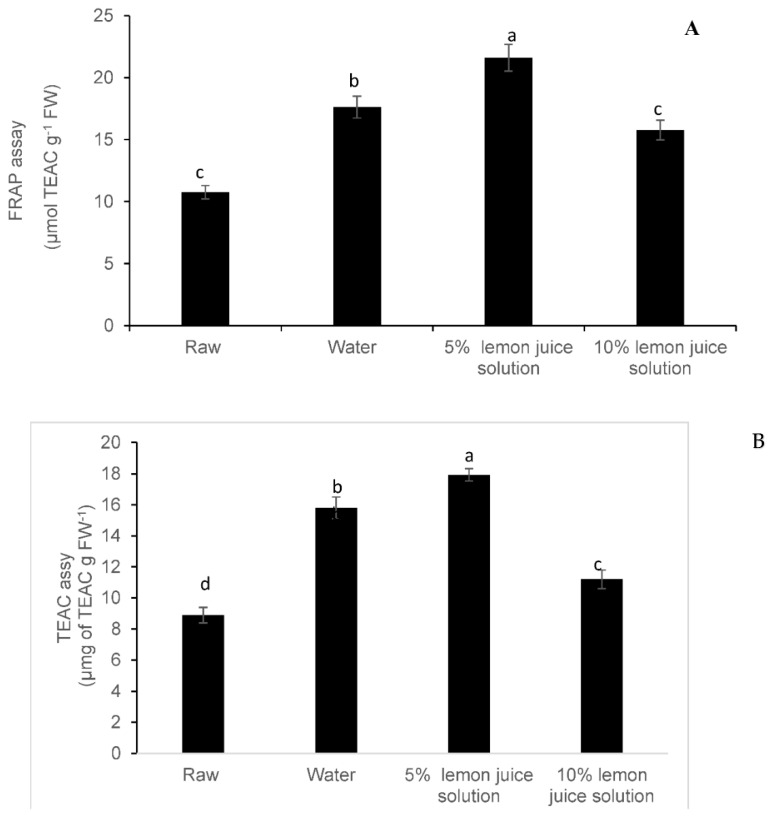
Effect of different types of moist cooking blanching treatments using a hot water bath at 95 °C on antioxidant capacity: (**A**) ferric reducing-antioxidant power assay (FRAP) and (**B**) Trolox equivalent antioxidant capacity (TEAC) assay in Chinese cabbage leaves. Bars with the same alphabetic letter for a specific phenolic compound are not significantly different at *p* < 0.05.

The FRAP and TEAC assays showed that the leaf samples blanched with 0.5% lemon water as a blanching medium had the strongest antioxidant capacity compared with the other samples and the raw leaves (Figure 6A,B). At the same time, the lowest antioxidant activity was noted in the unblanched leaf samples (raw leaves) (Figure 6). Moreover, the antioxidant capacity of the samples blanched with 10% lemon juice medium was the lowest among the three tested blanching treatments (Figure 6A,B).

**Figure 7:**
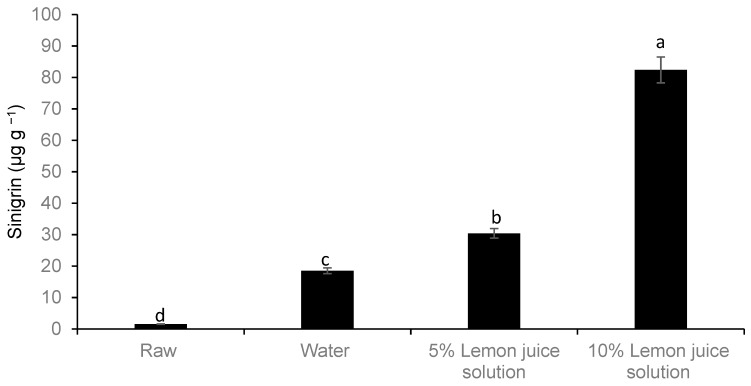
Effect of different types of moist cooking blanching treatments using a hot water bath at 95 °C on the concentration of predominant glucosinolate sinigrin. Bars with the same alphabetic letter are not significantly different at *p* < 0.05.

The concentration of glucosinolate sinigrin in freshly harvested Chinese cabbage (*Brassica rapa* L. subsp. *chinensis*) was almost 1.5 µg g^−1^ (Figure 7). The concentration significantly increased with the concentration of lemon juice (lower pH) at 95 °C (Figure 7). Furthermore, 1-Methoxy glucobrassicin was detected at lower concentrations than 0.1 µg g^−1^. However, 1-methoxy glucobrassicin increased up to 0.3 and 0.6 µg g^−1^ with increasing concentrations of lemon juice at 5% and 10%, respectively (data not presented). The total ion chromatogram in the ESI negative mode for glucosinolate sinigrin related to different blanching medium during hot water bath is given in Figure S2. 

## 4. Discussion

The primary parameter that determines the consumer purchasing power of a food product is colour [17]. Consumers like to purchase leafy vegetables that are fresh and green in colour. Green peppers blanched in lemon juice or vinegar lost their green colour and chlorophyll content due to the acidity of the treatments. However, it is important to note here that the increase in concentration of lemon juice in water played a major role in determining the colour change [18]. Also, the lower pH environment of the 20% lemon juice blanching medium would have facilitated the conversion of chlorophyll to pheophytins and was responsible for the loss of green colour as reported by Gunawan et al. [19]. The pheophytin and pheophorbide are produced because of the replacement of the magnesium ion in the porphyrin ring by hydrogen ions in the presence of a low pH medium [20]. Thus, the increase in colour difference was due to the change of the green colour to olive brown due to the formation of pheophytin and pheophorbide [20]. However, pheophytin was not quantified in this study. Furthermore, blanching inactivates the chlorophyllase enzyme responsible for the rapid degradation of the green colour [21]. Blanching in a hot water bath in 5% lemon juice acidic solution had improved the retention of total chlorophyll content mainly due to the improved extractability of the chlorophyll because of the matrix changes. The higher temperature, 95 °C, during hot water blanching could result in a greater rupturing of cell structure, which would have led to better solvent access and extraction [22]. A similar increase in chlorophyll content during blanching was reported in coriander leaves [23]. 

Thermal blanching treatments were shown to inactivate the polyphenol oxidase activity that uses the polyphenols as substrates for the browning reaction [24]. Also, the lower pH was shown to improve the extraction yield of phenolics [25], which could be responsible for the observed increase in total phenols compared to that of the raw Chinese cabbage leaves in this study (Figure 3). However, some researchers have shown a decrease in total phenolic compounds due to thermal degradation and leaching into the water [26]. The degree of the degradation of polyphenols depends on the processing time, heat and the portion size of the vegetables [27]. Some researchers have shown that warm treatments did not affect the level of polyphenols or kaempferol in onions, green beans and peas [28]. Furthermore, thermal treatments can inactivate the oxidative enzymes that are responsible for the oxidation of antioxidants and as a result can increase the antioxidant activity [29]. Also, according to the literature, thermal treatments have shown a significant increase in antioxidant activity in pepper, green beans, spinach, broccoli [30], tomato [31], broccoli cauliflower [32] possibly due to the increased discharge of antioxidant constituents from the matrix or the formation of redox-active secondary plant metabolites or breakdown products [33]. Furthermore, the total phenolic content and the antioxidant activity in sweet potato leaf polyphenols were higher in a neutral and weak acid pH solvent system and in neutral and weak acid solvent systems [34]. There was an increase in quinic acid in mild acidic condition with increasing temperature [34]. In this study, an increase in quinic acid in mild acidic conditions with increasing temperatures could have induced the significant changes of the total phenols [34]. Therefore, using a high acid concentration and thermal treatments must be avoided during the moist cooking of Chinese cabbage. Also, the strong acidic conditions and temperatures were reported to affect the quinic acid derivatives (esters) especially in chlorogenic acids where the reduce the number caffeoyl groups demonstrated a decreased antioxidant activity of molecule [35]. Quinic acid can be used as a flavour enhancer due to its characteristic astringent taste. Kaempferol glycosides were predominantly detected in Brassica cultivars. In Ethiopian kale (*B. carinta*), which is a popular indigenous vegetable in East Africa, belongs to the same Brassicaceae family and contains higher levels of coumaroyl-glucoside and a higher number of kaemperol and isorhamnetin diglycosides [36]. However, in Chinese cabbage (*Brassica rapa* L. subsp. *chinensis*), isorhamnetin diglycosides were not detected. 

Cauliflower blanched in water at 100 °C for 3 min was reported to reduce the total kaempferol content [37]. An increase in the concentration of the lemon juice (acidic conditions) at 95 °C affected the changes in the concentrations of the different kaempferol glycosides (kaempferol-dihexoside, kaempferol hexoside) and some are glycosylated with sophorotrioses—the kaempferol *O*-sophoroside-*O*-hexoside and myrectin-*O*-arabinoside in this study. A catalytic reverse shift reaction of kaempferol and myrectin glycosides or the release of bound phenolics into free phenolic derivatives [37] could possibly have taken place for the increase during blanching in 5% lemon solution at 95 °C. However, further investigations are needed to prove this hypothesis. Furthermore, the thermostability of flavonoids was reported to be dependent on their glycosylation and acylation status and an increase in non-acylated quercetin compounds was shown during thermal processing especially baking [38]. Also, the disappearance of protocatechuoyl hexose blanching treatment (heat) could possibly have been due to the ruptured phenol–sugar bond and resulted in the formation of the simple phenolic structure of the aglycone [38].

Similarly, an increase in quercetin-4′-*O*-monoglucoside and quercetin-3′-*O*-diglucoside in onions were shown to increase at higher temperatures, at 120 °C, in different onion cultivars—Colossal, Sunpower, Chairman, and 110,455 [39]. A low acidic medium during food processing that was reported to increase the flavone glycosides and the conversion of apiin to apigenin 7-*O*-glucoside in celery juice was reported at pH 5 [40]. It is important that the moist cooking blanching at pH 5 could facilitate the increase in kaempferol glycosides and potentially modulate its intestinal absorption and metabolism [40]. However, further investigations are needed to confirm its bioavailability for intestinal absorption. Kaempferol has shown numerous health benefits, mainly with anticarcinogenic, antiinflammatory, anti-obesity, and antiviral properties and its activities [38].

The total glucosinolate content in different cauliflower varieties such as cv. ‘Aviso’, ‘Dania’ (white varieties), ‘Grafitti’ (purple), ‘Emeraude’ (green) and ‘Celio’ (green pyramidal) were lost by 55% and 42% during blanching and boiling, respectively [41]. Similarly, in red cabbage, Brassica oleracea L. ssp. capitataf. rubra cv. ‘Autoro’, the glucosinolate content was reduced by 64%, 38% and 19% during blanching, boiling and steaming respectively [42]. However, steaming showed the least effect on the antioxidant constituents in cauliflower varieties [41]. Probably during blanching or boiling at higher concentrations, functional compounds are leached into the processing water [41]. Chinese cabbage (*Brassica rapa* L. subsp. *chinensis*) contains sinigrin as the predominant glucosinolate in this study. On the one hand, the blanching treatment was reported to reduce the total glucosinolate content of cabbage and 53% loss of sinigrin was reported during cooking, mainly due to leaching effects [43]. The blanching treatment was reported to reduce the total glucosinolate content of cabbage and a 53% loss of sinigrin was reported during cooking, mainly due to leaching effects [43]. On the other hand, the thermal process was suggested to reduce the formation of isothiocyanate, which possesses many health benefits such as antimicrobial, anti-inflammatory, antithrombotic and chemopreventive effects [44]. Ethopian kale (*B. carinata*) contains higher concentrations of glucosinolate aliphatic alkenyl glucosinolate 2-propeny, which has a chemo preventive property [43]. Therefore, the pH and temperatures during the different food preparation methods are important to maintain the biologically active isothiocyanate. In addition, the highest activity of myrosinase was reported in broccoli at pH 6.5–7.6 and in Brussels sprouts at pH 8 [45]. At the same time, the optimum temperature for myrosinase activity in broccoli and Brussels sprouts was reported to be 30 and 50 °C, respectively [45,46]. However, moist cooking blanching at 95 °C in 5% lemon juice could have inactivated the thermolabile endogenous myrosinase and the lower pH of the lemon solution could have prevented further hydrolysis during cell lysis in the process. However, further investigations are needed to elucidate the mechanism. Similarly, short blanching for 5 min in boiling water and fermentation with probiotic strain *Lactobacillus paracasei* LMG P22043 at a final pH of 4.12 reduced the loss of glucosinolates [43].

## 5. Conclusions

It is evident from this study that the moist cooking blanching affected the colour, chlorophyll content, phenolic and glucosinolate components and antioxidant properties. Moist cooking blanching using 20% lemon water significantly affected the colour, chlorophyll content, phenolic component and sinigrin glucosinolates and the antioxidant properties. However, moist cooking blanching using 5% lemon water significantly retained the colour and chlorophyll content and increased the concentration of kaempferol glycosides, gluconic acid, sinigrin glucoside and antioxidant activity. Further investigations are needed to explain the changes in the concentrations of kaempferol derivatives during moist cooking blanching using 5% lemon water and to investigate the biological effects. 

## Figures and Tables

**Table 1:** Tentative peak assignment of the metabolites contained in Chinese cabbage leaves subjected to moist cooking blanching treatments using different types of blanching medium.

Retention Time	M−H	M−H Formula	Error (ppm)	MSE Fragments	UV	Tentative Identification
0.8	195.0493	C_6_H_11_O_7_	−6.2		227	Gluconic acid
0.8	133.0127	C_4_H_4_O_5_	−7.5			Malic acid
0.92	191.0181	C_6_H_7_O_7_	0.6	155.127,111	280	Quinic acid
2.464	315.0707	C_13_H_15_O_9_	−2.9	153.109	306	Protocatechuoyl-hexose
2.72	771.1898	C_33_H_39_O_21_	−0.6	609.285	265.347	Kaempferol 3-*O*-sophoroside 7-*O*-hexoside
3.08	609.1463	C_27_H_29_O_16_	1.1	447.285	265.341	kaempferol-dihexoside
3.47	431.1916	C_20_H_31_O_10_	−0.2	385.153,97	330	Unknown
3.6	609.1488	C_27_H_29_O_16_	5.3	285.255	264.340	Kaempferol 3-*O*-sophoroside
4.14	447.0947	C_21_H_19_O_11_	4.2	285.255,99	264.350	Kaempferol hexoside
4.3	449.0743	C_20_H_17_O_12_	5.1	363.157,97	364.350	Myricetin 3-*O*-arabinoside
5.89	269.0488	C_15_H_10_O_5_	−0.2	151.133,119,97	262	Apigenin
6.37	327.2166	C_18_H_31_O_5_	1.5	229. 211,171,97	weak	unknown
6.85	329.2328	C_18_H_33_O_5_	0.2	211.171,97	270	unknown
7.73	307.191	C_18_H_27_O_4_	0.3	235.121	311	unknown
7.91	307.1913	C_18_H27O4	1.3	235.220,121,99	240	unknown
8.25	305.1747	C_18_H_25_O_4_	−2.6	249. 135	319	unknown
9.90	291.1958	C_18_H_27_O_3_	−0.7	277.265,121	280	unknown
10.37	293.211	C_18_H_29_O_3_	−2.4	255.185,143	280	unknown
12.07	591.2595	C_34_H_39_O_9_	0.2	515.325,183,149	409	unknown

**Table 2:** Exact mass/retention time pairs responsible for the separation of raw Chinese cabbage leaves.

	Retention Time	Mass	P(1)P	p(corr)(1)P
9.92_291.1957	9.92	291.1957	0.237452	0.971414
10.38_293.2110	10.38	293.211	0.2061	0.995988
8.27_305.1753	8.27	305.1753	0.231893	0.998607
7.74_307.1916	7.74	307.1916	0.168101	0.991243
2.46_315.0707	2.46	315.0707	0.2004	0.998301
6.37_327.2171	6.37	327.2171	0.237674	0.87965
6.85_329.2318	6.85	329.2318	0.146947	0.969433
3.85_385.1121	3.85	385.1121	0.166316	0.981166
3.47_431.1912	3.47	431.1912	0.19889	0.989573
3.47_483.1625	3.47	483.1625	0.13382	0.953158
10.88_555.2839	10,88	555.2839	0.175476	0.902287

**Table 3:** Exact mass/retention time pairs responsible for the separation of all hot water-blanched Chinese cabbage leaves irrespective of the type of blanching medium.

Primary ID	Retention Time	Mass	P(1)P	p(corr)(1)P
4.02_193.0497	4.02	193.0497	−0.21239	−0.912351
4.01_223.059	4.01	223.0599	−0.29435	−0.984697
3.48_325.0552	3.48	325.0552	−0.15576	−0.984697
4.01_339.0712	4.01	339.0712	−0.19615	−0.984697
2.90_963.2412	2.90	963.2412	−0.25714	−0.984697
3.13_977.2561	3.13	977.2561	−0.16666	−0.984697

**Table 4:** Effect of different types of moist cooking blanching treatments using hot water bath blanching at 95 °C on predominant phenolic compounds in Chinese cabbage leaves.

	Quinic Acid	Protocatechuoyl Hexose	Kaempferol *O*-Sophoroside-*O*-Hexoside (mg kg^−1^)	Kaempferol-Dihexoside	Ferulic Acid	Kaempferol-Sophoroside	Kaempferol Hexoside	Myrectin-*O*-Arabinoside
Raw	209.8 ± 0.02 ^c,^*	46.0 ± 0.07 ^a^	50.4 ± 0.01 ^a^	27.7 ± 0.05 ^b^	8.0 ± 0.03 ^c^	20.8 ± 0.02 ^b^	20.3 ± 0.06 ^b^	13.4 ± 0.03 ^a^
Water	709.7 ± 0.04 ^b^	0.0 ± 0.00 ^b^	31.8 ± 0.06 ^c^	19.9 ± 0.08 ^c^	462.9 ± 0.10 ^a^	28.9 ± 0.05 ^b^	28.9 ± 0.03 ^b^	8.5 ± 0.01 ^b^
5% lemon juice solution	765.9 ± 0.01 ^b^	0.0 ± 0.00 ^b^	46.4 ± 0.04 ^b^	37.1 ± 0.0l ^a^	463.9 ± 0.04 ^a^	73.6 ± 0.08 ^a^	69.9 ± 0.02 ^a^	6.9 ± 0.07 ^c^
10% lemon juice solution	1067.2 ± 0.05 ^a^	0.0 ± 0.00 ^b^	17.2 ± 0.03 ^d^	12.9 ± 0.04 ^d^	101.4 ± 0.03 ^b^	1.7 ± 0.03 c	0.8 ± 0.12 ^c^	2.1 ± 0.02 ^d^

Means with the same alphabetic letter for a specific phenolic compound are not significantly different at *p* < 0.05, * standard deviation.

## Supplementary Materials

The following are available online at https://www.mdpi.com/2304-8158/8/9/399/s1. Figure S1: Comparison of UPLC–Q-TOF/MS chromatogram illustrating the changes in phenolic compounds in (**A**) Raw Chinese cabbage leaves, blanching treatment using hot water bath at 95 °C using (**B**) 5% lemon juice as (**C**) using water as blanching (**D**) 10% lemon juice as blanching medium. The chromatograms of three replicates of each treatment (raw sample, 5% lemon juice, water, 10% lemon juice) were included. The relative peak intensity is normalized, and peaks are expressed as the percentage highest peak intensity; Figure S2: Comparison of UPLC–Q-TOF/MS chromatogram illustrating the changes in sinigrin (aliphatic glucosinolate) in (**A**) Raw Chinese cabbage leaves, blanching treatment using hot water bath at 95 °C using (**B**) 5% lemon juice as (**C**) using water as blanching (**D**) 10% lemon juice as blanching medium. The relative peak intensity is normalized, and peaks are expressed as the percentage highest peak intensity.
