# Supplementary material for: Effect of Moist Cooking Blanching on Colour, Phenolic Metabolites and Glucosinolate Content in Chinese Cabbage (Brassica rapa L. subsp. chinensis)
Source: Foods. 2019 Sep 8;8(9):399. doi: 10.3390/foods8090399 (PMC6770643; doi:10.3390/foods8090399)
Supplement: Supplementary file 1 [file foods-08-00399-s001.pdf]

Raw Chinese cabbage leaves

A

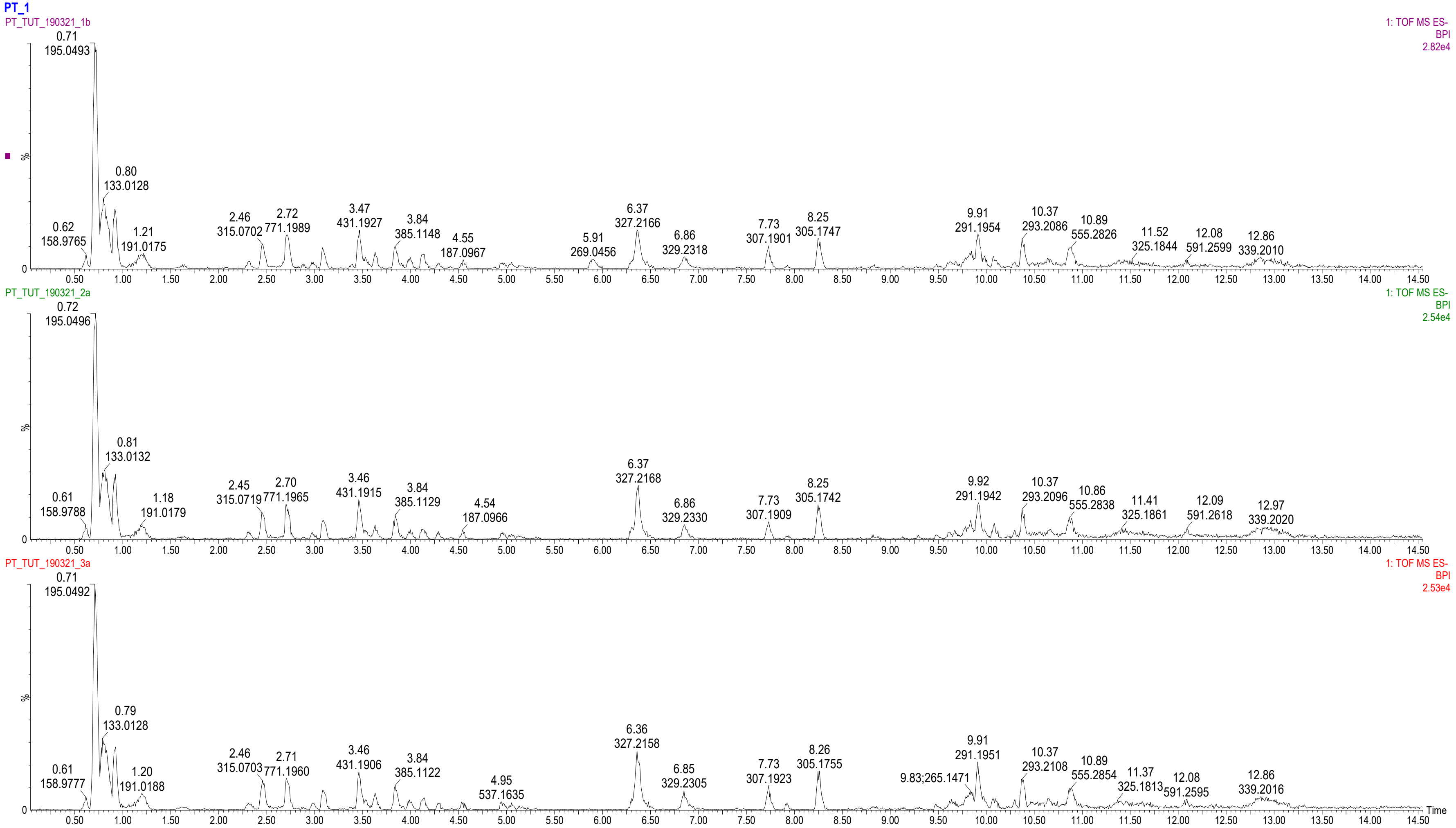

B

Hot water bath blanching using 5% lemon juice as blanching medium at 95 °C

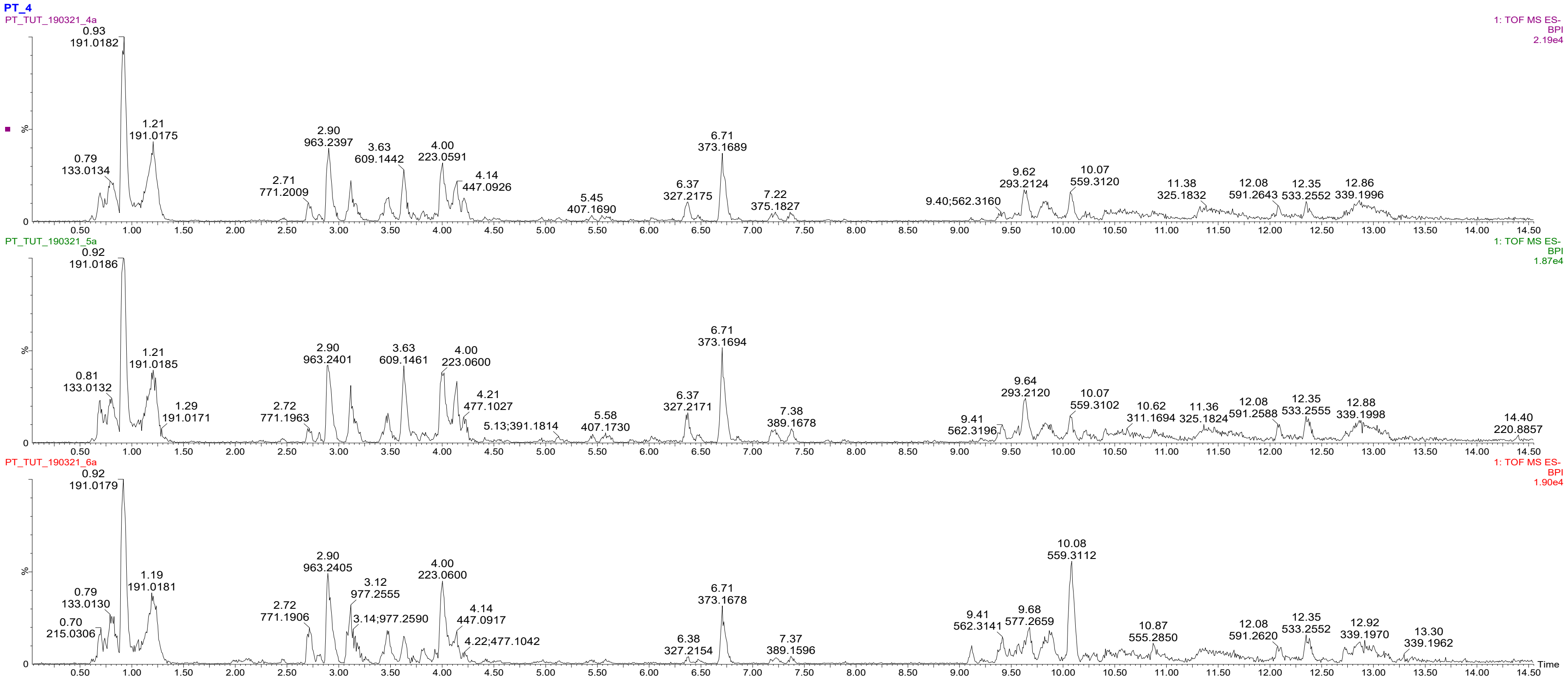

C

Hot water bath blanching using water as blanching medium at 95 °C

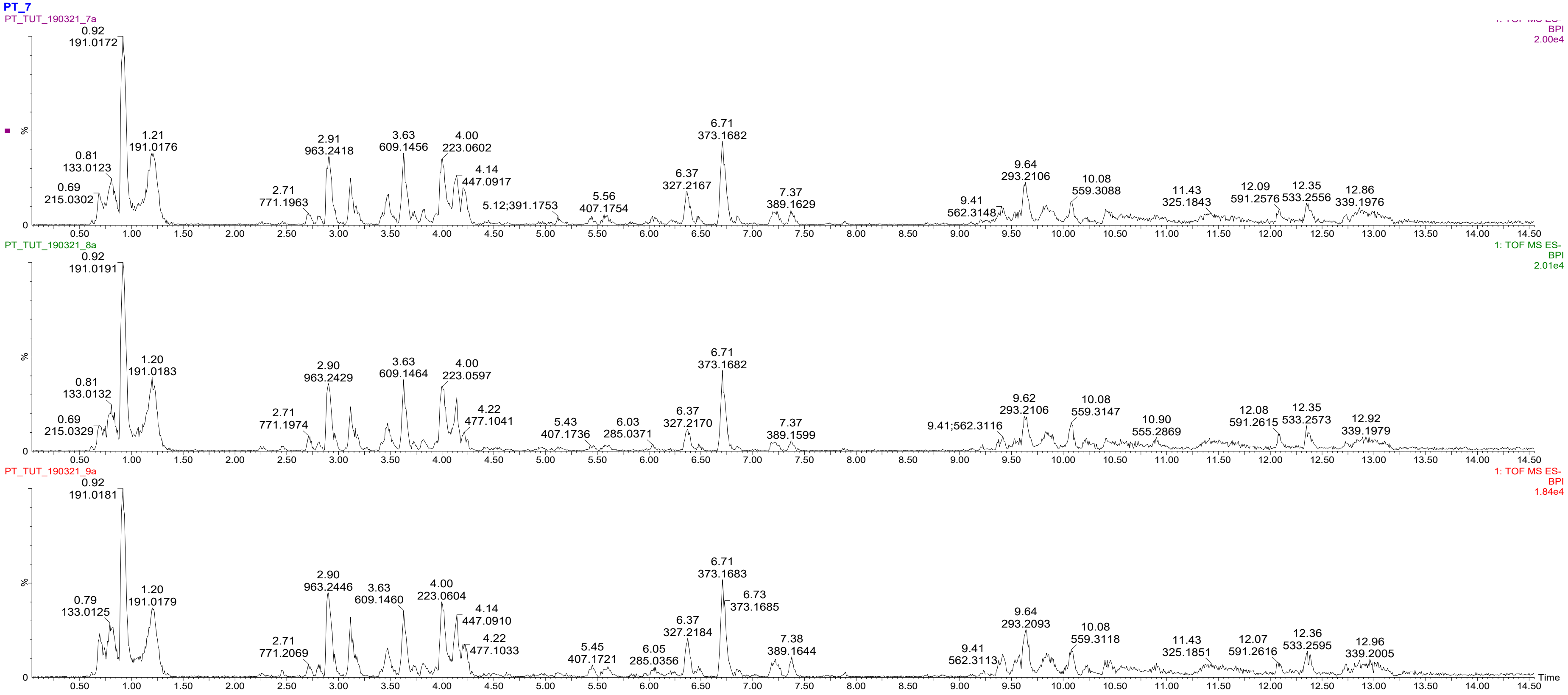

D

Hot water bath blanching using 10% lemon juice as blanching medium at 95 °C

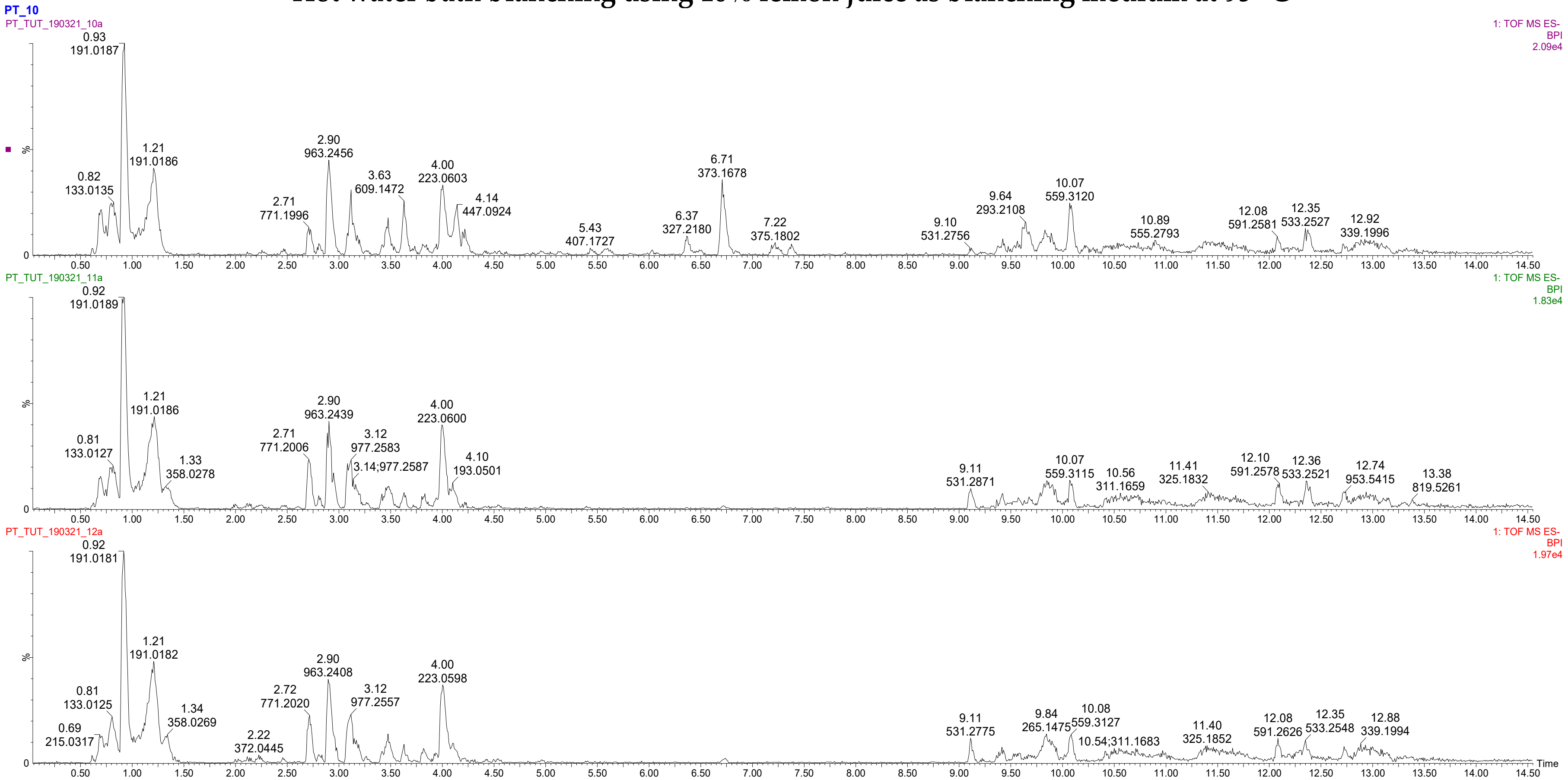

Figure S1. Comparison of UPLC–Q-TOF/MS chromatogram illustrating the changes in phenolic compounds in (**A**) Raw Chinese cabbage leaves, blanching treatment using hot water bath at 95 °C using (**B**) 5% lemon juice as (**C**) using water as blanching (**D**) 10% lemon juice as blanching medium. The chromatograms of three replicates of each treatment (raw sample, 5% lemon juice, water, 10% lemon juice) were included. The relative peak intensity is normalized, and peaks are expressed as the percentage highest peak intensity

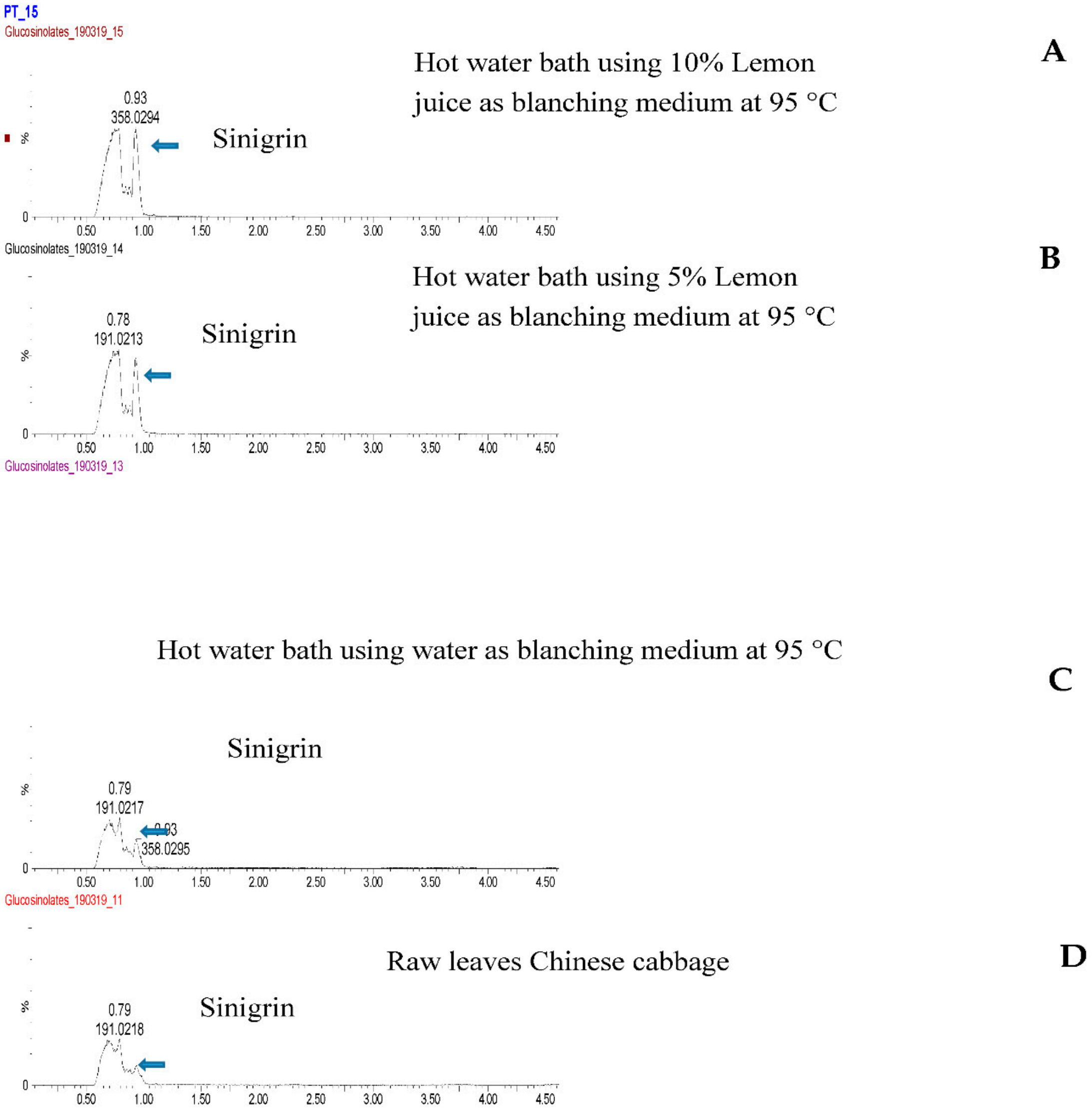

Figure S9. Comparison of UPLC–Q-TOF/MS chromatogram illustrating the changes in sinigrin (aliphatic glucosinolate) in (**A**) Raw Chinese cabbage leaves, blanching treatment using hot water bath at 95 °C using (**B**) 5% lemon juice as (**C**) using water as blanching (**D**) 10% lemon juice as blanching medium. The relative peak intensity is normalized, and peaks are expressed as the percentage highest peak intensity.
